# Active and machine learning-enhanced discovery of new FGFR3 inhibitor, Rhapontin, through virtual screening of receptor structures and anti-cancer activity assessment

**DOI:** 10.3389/fmolb.2024.1413214

**Published:** 2024-06-11

**Authors:** Qingxin Zeng, Haichuan Hu, Zhengwei Huang, Aotian Guo, Sheng Lu, Wenbin Tong, Zhongheng Zhang, Tao Shen

**Affiliations:** ^1^ Department of Thoracic Surgery, Sir Run Run Shaw Hospital, Zhejiang University School of Medicine, Hangzhou, China; ^2^ Department of Thoracic Surgery, Longyou County People’s Hospital, Hangzhou, China; ^3^ Department of Emergency Medicine, Sir Run Run Shaw Hospital, Hangzhou, China

**Keywords:** NSCLC, FGFR3 inhibitor, AI-derived drug discovery, Rhapontin, biology evaluation

## Abstract

**Introduction:** This study bridges traditional remedies and modern pharmacology by exploring the synergy between natural compounds and Ceritinib in treating Non-Small Cell Lung Cancer (NSCLC), aiming to enhance efficacy and reduce toxicities.

**Methods:** Using a combined approach of computational analysis, machine learning, and experimental procedures, we identified and analyzed PD173074, Isoquercitrin, and Rhapontin as potential inhibitors of fibroblast growth factor receptor 3 (FGFR3). Machine learning algorithms guided the initial selection, followed by Quantitative Structure-Activity Relationship (QSAR) modeling and molecular dynamics simulations to evaluate the interaction dynamics and stability of Rhapontin. Physicochemical assessments further verified its drug-like properties and specificity.

**Results:** Our experiments demonstrate that Rhapontin, when combined with Ceritinib, significantly suppresses tumor activity in NSCLC while sparing healthy cells. The molecular simulations and physicochemical evaluations confirm Rhapontin’s stability and favorable interaction with FGFR3, highlighting its potential as an effective adjunct in NSCLC therapy.

**Discussion:** The integration of natural compounds with established cancer therapies offers a promising avenue for enhancing treatment outcomes in NSCLC. By combining the ancient wisdom of natural remedies with the precision of modern science, this study contributes to evolving cancer treatment paradigms, potentially mitigating the side effects associated with current therapies.

## 1 Introduction

Lung cancer, in its various forms, poses a severe global health challenge. Accounting for approximately 85% of all lung cancer cases, non-small cell lung cancer (NSCLC) reigns as the most prevalent form of this malignancy (Sung et al., 2021). As per recent global statistics, NSCLC maintains a distressingly high mortality rate, cementing its position as one of the leading contributors to cancer-related deaths worldwide (Siegel et al., 2024). Concurrently, the incidence rate for NSCLC continues on an upward trend. Despite significant strides in diagnostic technologies and therapeutic methodologies, the prognosis for NSCLC remains bleak, with a 5-year survival rate barely reaching the 20% threshold (de Groot et al., 2018). The persistence of this grim statistic highlights the urgency for development of more effective therapeutic strategies in the battle against NSCLC.

A potentially promising approach in ameliorating treatment efficacy involves sensitizing cancer cells to extant therapeutic agents. Amidst the plethora of targets, the spotlight has recently shifted towards the Fibroblast Growth Factor Receptor 3 (FGFR3) (Turner and Grose, 2010). FGFR3, part of the larger fibroblast growth factor receptor family, has been implicated in numerous cellular processes, including cell proliferation, survival, and differentiation (Wesche et al., 2011). Recent studies suggest that modulation of FGFR3 activity could potentially augment the effectiveness of existing treatments, such as Ceritinib—an ALK (Anaplastic Lymphoma Kinase) inhibitor (Tang et al., 2008; Zhang et al., 2013). Despite this promising insight, therapeutic combinations incorporating FGFR3 inhibition to augment sensitivity of NSCLC cells to Ceritinib remain largely unexplored.

Historically, the drug development process has heavily leaned on experimental methodologies. Whilst these approaches have their merit, they come saddled with a suite of limitations. For instance, these traditional strategies often prove to be labor-intensive and time-consuming (Munos, 2009). Moreover, their applicability to high-throughput screening is limited, thereby underscoring the need for more efficient methodologies (Paul et al., 2010). Enter the realm of computational biology, which offers a more expedient alternative to traditional strategies. With the ability to conduct *in silico* screenings of expansive compound libraries, computational approaches promise significant savings in terms of both time and resources (Ekins et al., 2007; Green, 2008). These techniques enable prediction of interactions between small molecules and protein targets, thus providing preliminary insights into the potential efficacy and toxicity of candidate inhibitors. Complementing this, molecular dynamics (MD) simulations furnish a more granular understanding of the behaviour of protein-ligand complexes over time, thereby enhancing our grasp of the binding process (Dror et al., 2012; Arnittali et al., 2019).

Herein, we propose a melding of virtual screening and MD simulations as an integrative approach to identifying prospective FGFR3 inhibitors. Our overarching goal is to enhance the sensitivity of NSCLC cells to Ceritinib, offering a potentially viable strategy to circumvent the common therapeutic resistance observed in NSCLC. This innovative methodology presents a novel angle to the design of inhibitors, potentially paving the way for breakthrough combination therapies for NSCLC (Brown and Toker, 2015; Colmegna et al., 2018). By boosting the efficacy of treatment regimens, such therapeutic strategies have the potential to significantly enhance the prognosis for NSCLC patients, impacting a large patient population worldwide.

## 2 Method

### 2.1 Structure relaxation

In the pursuit of FGFR3 inhibitors, structure-based computational methodologies were employed, utilizing the FGFR3 crystal structure (PDB code: 6LVM) in complex with Pyrimidine Derivative 37b was selected as the receptor protein (Kuriwaki et al., 2020). All molecular dynamics (MD) simulations presented in this study were conducted using the GROMACS 23.1 package (https://www.gromacs.org/). The AMBER 99SB-ILDN (Lindorff-Larsen et al., 2010) and explicit solvation were employed, and each system was placed in a rectangular box of SPC water molecules with a minimum distance of 10Å between any solute atom and the edges of the periodic box. Counter ions were added to neutralize the total charge of the system. The system underwent an energy minimization process using the steepest descent method, with the maximum set to 1000.0 kJmol^−1^nm^−1^. Subsequently, the system was equilibrated in two steps: 1) canonical ensemble (NVT, 1ns) and 2) isothermal–isobaric ensemble (NPT, 1ns). Following equilibration, the MD simulations were run for 500ns. To ensure numerical stability, all bonds involving hydrogen atoms were constrained using the default linear constraint solver algorithm (LINCS) (Hess, 2008). The Vrescale thermostat and Parrinello–Rahman barostat were utilized with the temperature set at 300 K and pressure at 1.0bar, with time constants of 0.1 and 2ps, respectively. The Particle-Mesh Ewald (PME) method was employed to handle long-range interactions, and a 10Å cutoff was utilized for van der Waals interactions (Darden et al., 1993). The time step was set to 2 fs, and a snapshot was collected every 1.0 ps The free energy landscape (Malmstrom et al., 2015) was obtained by means of covariance matrix construction and principal component analysis (PCA) (Campitelli et al., 2021) to explore the local conformational landscape and return to a local energy minimum.

### 2.2 Protein preparation

The Schrödinger Protein Preparation Wizard was employed to meticulously prepare the complex, involving various steps such as adding missing hydrogen atoms, correcting metal ionization states, enumerating bond orders in HET groups, determining ligand protonation states and associated energy penalties, optimizing histidine residues’ protonation states, rectifying potentially transposed heavy atoms, optimizing the protein’s hydrogen bond network, and performing a restrained minimization. The binding region within the 3D receptor structure, where the Pyrimidine Derivative 37b binds, was identified as the screening ligands’ target site, and a corresponding grid was created.

### 2.3 Active learning based virtual screening

Active Learning Glide will generate a receptor grid from a prepared protein and prepare the TargetMol Natural Compound Library, which contains approximately 190,000 compounds. All of these compounds are available for purchase. It will also dock a subset of these ligands using Glide SP (Friesner et al., 2004). Active Learning workflows train a machine learning (ML) model on physics-based data, such as FEP+(Wang et al., 2015) predicted affinities or Glide docking scores, iteratively sampled from a full library using Schrödinger’s deep-learning powered QSAR platform, DeepAutoQSAR (https://www.schrodinger.com/science-articles/benchmark-study-deepautoqsar-chemprop-and-deeppurpose-admet-subset-therapeutic-data). 3 iterative training rounds were set. After all the ligands have been screened using the last model, a selection of the top ligands will then be docked using Glide SP.

### 2.4 Machine learning principles using AutoQSAR

AutoQSAR is a machine-learning algorithm provided by the Schrödinger suite that builds and applies QSAR models through automation (Dixon et al., 2016). In order to build a predictive model, AutoQSAR takes the one-, two-, and three-dimensional structural data of a molecule along with a IC_50_ property to be modeled as an input. It will then compute the fingerprints and descriptors, using machine-learning statistical methods to create a predictive QSAR model. The process utilizes multiple regression algorithms, including optimal subset multiple linear regression (MLR), partial least squares regression (PLS), kernel-based least squares regression (KPLS), and principal component regression (PCR), to construct numerical models. The predictive accuracy of the model is evaluated using various parameters such as ranking score, root mean square error (RMSE), standard deviation (SD), Q^2^, and *R* ^2^ values (de Oliveira and Katekawa, 2018). It is worth mentioning that the present analysis utilizes a series of Pyrimidine Derivative 37b (Kuriwaki et al., 2020) and some clinically oriented medicines from Drugbank for predictive model development.

### 2.5 Binding pose metadynamics

The metadynamics simulations employed a hill height of 0.05 kcal/mol and a width of 0.02 Å. RMSD calculations were performed by considering a distance of 3 Å between protein residues and ligands. Prior to the metadynamics simulations, the system underwent preparation in an SPC water box, followed by energy minimization, constraint application, and a gradual temperature increase to 300 K. The last 0.5 nanoseconds of an unbiased MD simulation served as the reference for the subsequent metadynamics protocol.

Three BPMD scores, namely, PoseScore, PersScore, and CompScore, were utilized to assess the stability of ligand binding. PoseScore represented the average RMSD from the ligand’s initial pose, where a steeper increase indicated instability in ligand binding. A PoseScore below 2 Å was considered indicative of a stable ligand-protein complex (Fusani et al., 2020). PersScore quantified the persistence of hydrogen bonds (HB) during the metadynamics simulations, with higher values indicating greater stability. Finally, the CompScore, a composite score, was obtained by linearly combining the PoseScore and PersScore (Jin et al., 2023). Lower CompScore values were associated with more stable ligand-protein complexes.

### 2.6 Physicochemical property and medicinal chemistry property prediction

The most promising compounds, identified through structure-based virtual screening, underwent further evaluation using ADMETlab 2.0 (Xiong et al., 2021). The analysis aimed to provide valuable insights into the compounds’ pharmacokinetic properties, bioavailability, and overall suitability as potential drug candidates.

### 2.7 Molecular dynamic simulation of desmond

In the initial phase, all-atom molecular dynamics (MD) simulations were conducted using the Desmond module of the Schrödinger software package. The simulations were performed within Maestro, starting with docked complexes that were placed in a cubic water box with a buffer distance of 10 Å. The systems were solvated with SPC water models, and a 0.15 M NaCl salt concentration was introduced for physiological relevance. To maintain system neutrality, additional Na^+^ and Cl^−^ ions were included. Long-range electrostatic interactions were computed using the particle-mesh Ewald method, while short-range van der Waals and Coulomb interactions were cutoff at 9.0 Å.

Following solvation, the systems underwent minimization and equilibration using the default Desmond protocol in Maestro. This involved restrained simulations in both the NVT (constant number of particles, volume, and temperature) and NPT (constant number of particles, pressure, and temperature) ensembles. After equilibration, a 100 ns MD simulation was performed in the NPT ensemble with periodic boundary conditions. The OPLS4 force field was employed to describe interatomic interactions. The temperature was maintained at 300 K using the Nosè-Hoover chain thermostat, and the pressure was kept at 1 atm using the Martyna-Tobias-Klein barostat method.

### 2.8 Cell culture

Two distinct cell lines were employed for the experimentation: A549 cells, characterized as an adenocarcinoma human lung epithelial cell line, and BEAS-2B cells, identified as a human bronchial epithelial cell line. These cell lines were sourced from iCell Bioscience Inc. Located in Shanghai, China. Both A549 and BEAS-2B cell lines have been authenticated using short tandem repeat (STR) analysis. A549 cells were nurtured using Ham’s F-12K (Kaighn’s) medium, while BEAS-2B cells were cultivated in Dulbecco modified Eagle’s medium (DMEM). In both cases, the culture mediums were supplemented with 10% exosome-depleted fetal bovine serum (EXO-FBS-50A-1) from System Biosciences, Palo Alto, CA, to eliminate potential interference from bovine exosomes. Additionally, a 1% penicillin-streptomycin solution (Tianhang Biotechnology, Hangzhou, China) was added. The cells were incubated under controlled conditions at 37°C within a 5% CO_2_ atmosphere (Exosomes of A549 Cells Induced Migration, Invasion, and EMT of BEAS-2B Cells Related to let-7c-5p and miR-181b-5p).

### 2.9 Cell viability detected by CCK8

After co-cultured for 24 h, cell proliferation was detected with CCK8 detection kit. Each well was incubated with 10 μL CCK8 detection reagent at 37°C for 2 h. The OD value of each well was detected with the microplate reader at 450 nm wavelength to calculate cell viability.

## 3 Results

### 3.1 Relaxation of FGFR3 structure

During the virtual screening process, the identification of compounds with the closest and most stable interactions with the target is crucial to selecting potential drug candidates. Molecular dynamics simulations of the target’s lowest energy conformation offer valuable insights into compounds with favorable binding affinities, providing crucial guidance for subsequent experimental screenings. To achieve this, a 500 ns molecular dynamics simulation was performed to explore FGFR3’s lowest energy conformation after releasing the Pyrimidine Derivative 37b, ensuring comprehensive sampling and equilibrium attainment for subsequent pocket-based virtual screenings.

**FIGURE 1:**
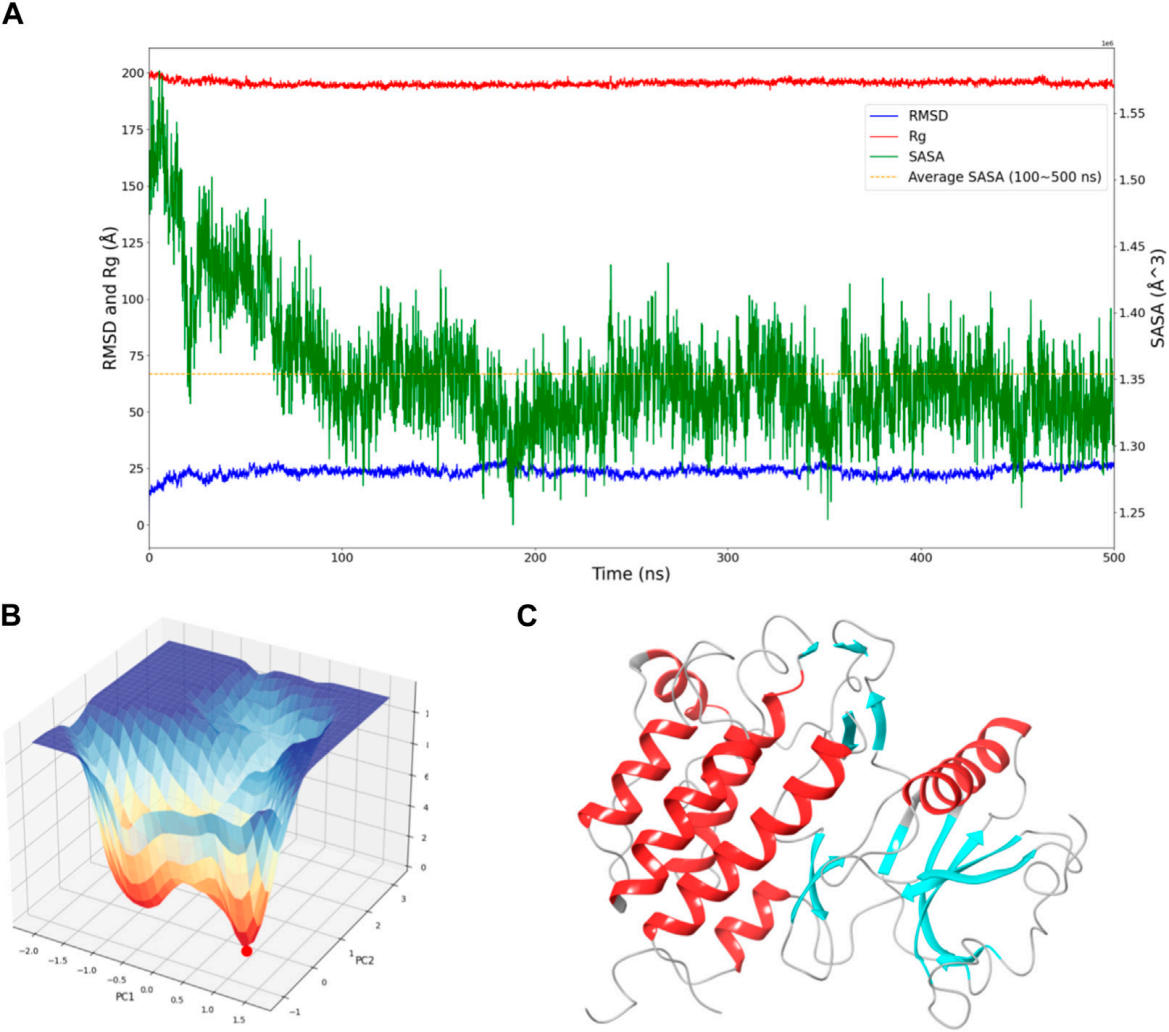
Dynamic Behavior and Free Energy Landscape of FGFR3. **(A)** The graph displays the time-dependent dynamics of FGFR3, including RMSD and Rg shown on the left *y*-axis, and SASA shown on the right *y*-axis. The dashed line represents the average SASA value after a sharp decrease. **(B)** The 3D free energy landscape of FGFR3 is depicted, with the energy minima indicated by the red dot. A 2D projection of the landscape provides an overview of the conformational space explored by FGFR3. **(C)** Resting state of FGFR3.

To assess the convergence of the simulation, RMSD, Rg, and SASA of FGFR3 were calculated. As shown in Figure 1A, during the 500 ns simulation, both the RMSD and Rg of FGFR3 exhibited minimal fluctuations, indicating an early attainment of stability. Regarding Figure 1A, the high RMSD observed likely results from significant conformational changes in the FGFR3 protein following the removal of the ligand from its binding site.While SASA showed some dynamic changes, it oscillated around the average value after 100 ns, suggesting a continuous periodic thermal motion of FGFR3 rather than a lack of equilibrium. Based on these parameters, the system was considered to reach equilibrium and achieve thorough sampling of FGFR3 after releasing the Pyrimidine Derivative 37b.

Subsequently, Gibbs free energy was statistically analyzed during the simulation, and a free energy landscape was constructed using the first and second eigenvectors, as shown in the Figure 1B. Three energy basins were identified, with the highest energy basin corresponding to the state when FGFR3 was bound to the Pyrimidine Derivative 37b, and the lowest energy points distributed in the remaining two smaller-volume basins. The transition state connecting these two states was determined. Notably, the lowest energy point emerged at 175 ns and remained stable until 500 ns, smoothly connecting the initial and final states. Based on this, we concluded that the 500 ns simulation successfully sampled FGFR3 after releasing the Pyrimidine Derivative 37b and the lowest energy point in the free energy landscape represented the resting state of FGFR3, as shown in Figure 1C. Building upon this information, subsequent pocket-based virtual screenings will be conducted.

### 3.2 Virtual compound screening and activity forecasting through active learning

Machine learning and deep learning have revolutionized drug discovery by powering applications such as structure-based virtual screening, efficiently sifting through compound libraries to identify potential hits, and activity prediction models leveraging molecular features to accurately estimate compound bioactivity.

**FIGURE 2:**
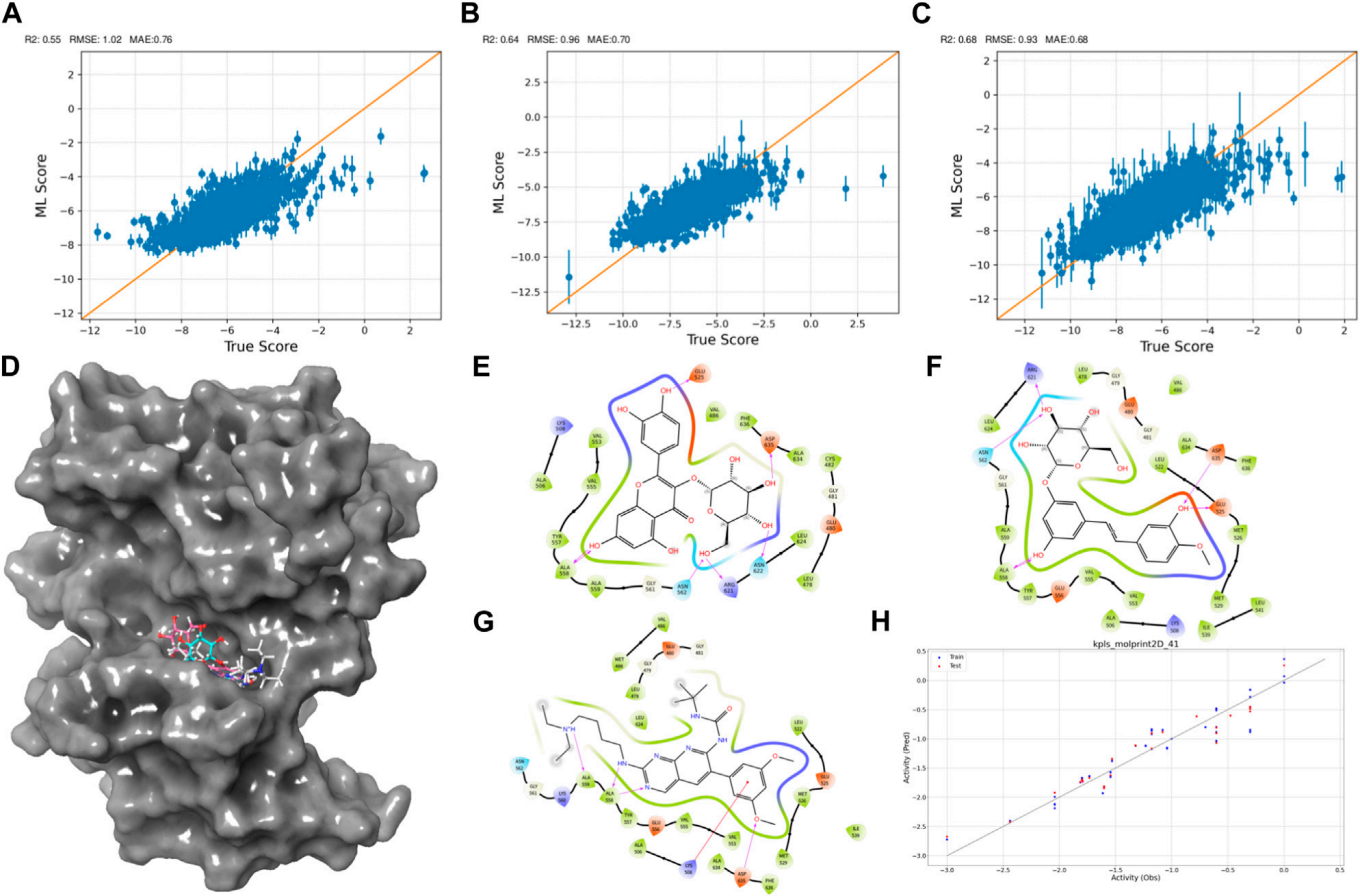
Active Learning-Assisted Virtual Screening and Activity Anticipation. **(A–C)** Three rounds of pre-training iterations for active learning docking. **(D)** Distribution of screened compounds on the surface of FGFR3. **(E–G)** 2D interaction plots of Rhapontin, Isoquercitrin, PD173074. **(H)** Evaluation of predictive performance of the QSAR model.

As the iterations progressed, the models consistently exhibited improved performance, as shown in Figures 2A–C. In the initial iteration, the coefficient of determination (*R* ^2^) value was 0.55, accompanied by root mean square error (RMSE) and mean absolute error (MAE) values of 1.02 and 0.7, respectively. Notably, the second iteration displayed an enhanced *R* ^2^ of 0.64, alongside reduced RMSE (0.96) and MAE (0.7) values, indicating improved model accuracy. The third iteration showed the most significant advancement, achieving an *R* ^2^ value of 0.68. Additionally, the RMSE decreased to 0.93, and the MAE reached 0.68, suggesting an increasingly precise parameter prediction.

Through meticulous analysis, incorporating a comprehensive evaluation of Docking Score, State Penalty, Ligand Strain Energy, and MMGBSA ΔG Bind, three compounds—PD173074 (Lamont et al., 2011), Isoquercitrin (Valentova et al., 2014), and Rhapontin—emerged as promising candidates. A detailed list of scores is presented in Table 1. PD173074 demonstrated exceptional binding affinity with a Docking Score of −10.3, and exhibited optimal receptor conformation with a State Penalty of 0, alongside a favorable Ligand Strain Energy of 2.1 kcal/mol and a MMGBSA ΔG Bind of −56.5 kcal/mol. Isoquercitrin also presented a strong case, with a Docking Score of −11.1, State Penalty of 0, Ligand Strain Energy of 9.1 kcal/mol, and a MMGBSA ΔG Bind of −94.4 kcal/mol. Rhapontin, while displaying a slightly higher Docking Score of −10.2, maintained a State Penalty of 0, a Ligand Strain Energy of 5.0 kcal/mol, and a MMGBSA ΔG Bind of −68.6 kcal/mol, ensuring its position as a candidate of interest. These stringent criteria ensured the selection of compounds not just with strong binding affinities, but also with optimal receptor conformations and stability, providing a solid foundation for the subsequent stages of our analysis and future experimental validation.

**TABLE 1:** Binding characteristics of tested compounds.

Name	Docking score	Glide ligand efficiency	MMGBSA dG bind	Lig strain energy
Forsythoside A	−16.884	−0.384	−49.03	47.778
Apigenin 7-O-(2G-rhamnosyl)gentiobioside	−15.204	−0.292	−75.5	18.267
Vitexin -4″-O-glucoside	−15.169	−0.361	−59.14	13.998
Kuromanin chloride	−14.644	−0.458	−56.76	22.869
Xylopentaose	−14.321	−0.311	−54.68	18.536
Neoeriocitrin	−14.184	−0.338	−69.72	21.13
Pectolinarin	−14.158	−0.322	−62.19	28.427
Isoquercitrin	−13.789	−0.418	−60.55	9.111
Gossypin	−13.72	−0.404	−72.69	6.723
Plantainoside D	−13.697	−0.304	−72.94	19.812
Neohesperidin	−13.535	−0.315	−79.03	15.221
Neodiosmin	−13.367	−0.311	−75.18	14.731
YKL-05–099	−13.297	−0.309	−80.06	11.416
Desmopressin	−13.228	−0.179	−64.58	32.245
Rhaponiticin	−13.204	−0.44	−69.41	4.781
Luteolin-7-glucuronide	−13.175	−0.399	−37.65	11.544
Homoplantaginin	−13.049	−0.395	−52.66	7.762
PD173074	−12.992	−0.342	−96.31	4.589
Didymin	−12.788	−0.304	−44.01	28.096
Pyrimidine derivative	−14.134	−0.267	−108.12	9.293

Then, we explored interactions between Isoquercitrin, PD173074, and Rhapontin with FGFR3 residues, as shown in Figures 2D–G. Crucial binding residues, such as Lys-508 (ATP-binding site) and Asp-617 (active site), were identified. Isoquercitrin engaged FGFR3 residues Ala-558, Ala-559, Lys-508, and Asp-635, indicating potential modulation of the ATP-binding pocket and its vicinity. PD173074s interactions encompassed Arg-621, Asn-562, Asp-635, Glu-525, and Ala-558, pointing to involvement with the active site and neighboring domains. Rhapontin’s interactions spanned Glu-525, Asp-635, Asn-622, Arg-621, Asn-562, and Ala-558, showcasing its adaptable binding capacity across critical regions.

Our Quantitative Structure-Activity Relationship (QSAR) modeling efforts yielded compelling results, as shown in Figure 2H. The training set exhibited a Q^2^ of 0.2402 and an *R* ^2^ of 0.8980, confirming the model’s ability to capture intricate activity relationships within the dataset. During external validation, the testing set demonstrated an RMSE of 0.2171 and a Q^2^ of 0.9069, attesting to the model’s robustness. Furthermore, the model demonstrated predictive prowess by estimating IC_50_ values for Isoquercitrin, Rhapontin, and PD173074. The calculated values—18.45 nM, 17.46 nM, and 11.67 nM—underscore the model’s potential to anticipate compound activities across different chemical entities. The notably close alignment between the predicted IC50 for PD Compound and experimental IC50 in the RT112 cell line targeting FGFR3 (Lamont et al., 2011) bolsters the model’s theoretical reliability.

### 3.3 Investigating binding mode and stability based on md simulation analysis for potential binding candidates

The results obtained from virtual screening required validation through molecular dynamics simulations to assess their dynamic behavior and interaction stability within the biological system, providing crucial theoretical guidance for further confirmation of potential drug candidates’ efficacy and safety in drug development.

**FIGURE 3:**
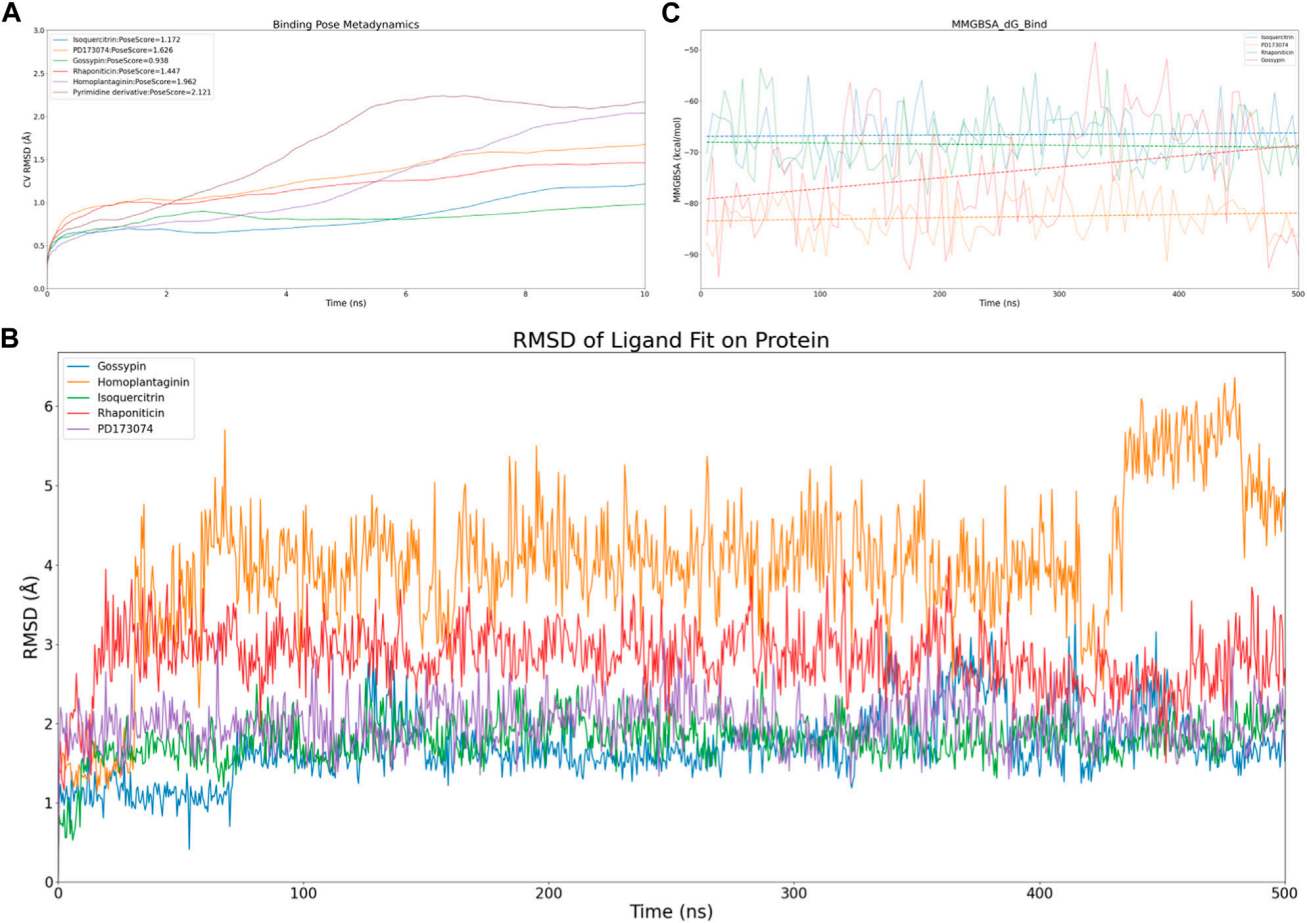
Virtual screening results rescreening based on molecular dynamics simulation. **(A)** Time-dependent CV RMSD Analysis of FGFR3 Complexes with Various Compounds. **(B)** Time-dependent RMSD of Ligand Fit on Protein Analysis of FGFR3 Complexes with Various Compounds. **(C)** Time-dependent MMGBSA of FGFR3 Complexes with Various Compounds.The solid lines of different colors represent the MMGBSA scores of different compounds interacting with FGFR3 at each time point. The dashed lines of different colors represent the average MMGBSA scores of different compounds over the 500 ns simulation.

To efficiently assess the stability of ligands in solution, we employed binding pose metadynamics (BPMD) as an enhanced sampling technique. By applying bias in the metadynamics simulation, ligand poses that exhibited instability were likely to be rarely occupied in the energy landscape, thereby exerting minimal influence on the overall binding affinity. We performed ten sets of BPMD simulations for the five compounds, with Pyrimidine Derivative 37b as a reference. The results, as shown in Figure 3A, indicated that the CV RMSD values remained below 2.5 Å for all five compounds, whereas only Pyrimidine Derivative 37b′s PoseScore exceeded 2 Å, suggesting that the remaining five compounds possessed stronger and more stable interactions with FGFR3 (Allegra et al., 2021). For a detailed list of scores, refer to Table 2.

**TABLE 2:** Dynamic interaction scores for tested compounds.

Compound name	PersScore	PoseScore	CompScore
Isoquercitrin	0.577	1.172	−3.828
PD173074	0.641	1.626	−3.374
Gossypin	0.348	0.938	−4.062
Rhaponiticin	0.43	1.447	−3.553
Homoplantaginin	0.265	1.962	−3.038
Pyrimidine derivative	0.752	2.121	−1.639

Despite conducting ten sets of simulations, the BPMD simulation time remained relatively short. Subsequently, we performed classical molecular dynamics simulations for the five compounds for an extended period of 500 ns, employing Ligand Fit on Protein RMSD and MMGBSA as reference values to evaluate the complex from both conformational and energetic perspectives. Ligand Fit on Protein RMSD represents the RMSD of a ligand when the protein-ligand complex is first aligned on the protein backbone of the reference, and then the RMSD of the ligand heavy atoms is measured. If the observed values are significantly larger than the RMSD of the protein, it suggests that the ligand may have diffused away from its initial binding site.

First, we evaluated the conformational changes, as shown in Figure 3B, which indicated that the five compounds exhibited a similar trend of achieving preliminary stability within the first 100 ns of the simulation. However, after 400 ns, Homoplantaginin showed a noticeable increase in RMSD, implying a potential time-dependence in its binding to FGFR3, and a possibility of off-target effects. Subsequently, we assessed the energetic aspects, focusing on the four remaining compounds since Homoplantaginin displayed potential off-target behavior. The MMGBSA results, as shown in Figure 3C, displayed significant fluctuations. To facilitate result analysis, we plotted the trendlines of four groups of MMGBSA values over time. The results revealed a clear upward trend for Gossypin, indicating a continuous decrease in binding energy between Gossypin and the receptor. This suggested that as the conformational adjustments continued, the binding energy between Gossypin and FGFR3 may decrease further, possibly leading to Gossypin dissociation from the binding pocket, implying the possibility of off-target effects. In summary, Isoquercitrin, Rhaponiticin, and PD173074 demonstrated a high potential to act as FGFR3 inhibitors, both from the conformational and energetic perspectives. Consequently, these three compounds were chosen for further interaction analysis.

### 3.4 Physicochemical parameters, medicinal chemistry parameters, and selectivity analysis

The physicochemical parameters and medicinal chemistry parameters of the compounds provided essential initial evaluations for drug development, aiding in the screening of potentially drug-like compounds. The selectivity analysis also contributed to identifying potential advantageous targets and guiding subsequent drug optimization and development, thereby increasing the likelihood of successful drug development.

**FIGURE 4:**
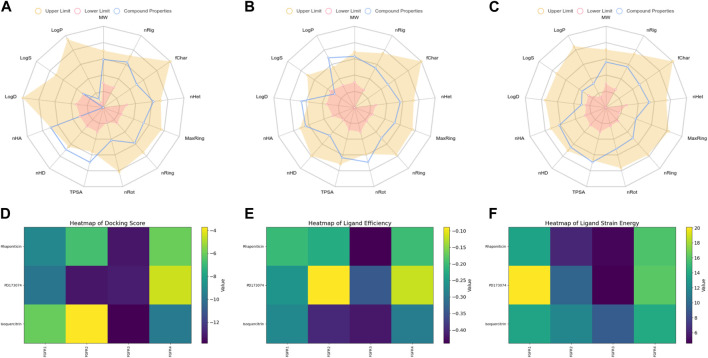
Physicochemical property and selectivity of 3 compounds.**(A)** Radar of Isoquercitrin physicochemical property.**(B)** Radar of PD173074 physicochemical property.**(C)** Radar of Rhaponiticin physicochemical property.**(D)** Docking scoring heatmap of three compounds with four FGFRs.**(E)** Ligand efficiency heatmap of three compounds with four FGFRs.**(F)** Ligand strain energy heatmap of three compounds with four FGFRs.

The radar plot in Figures 4A–C illustrates the analysis of physicochemical parameters, such as MW, TPSA, and LogP, for the investigated compounds. Rhapontin was the only compound that fell within the specified threshold range. The medicinal chemistry studies presented in Table 3 showed that Rhapontin exhibited a higher QED (Quantitative Estimate of Drug-likeness) (Kosugi and Ohue, 2021) value, indicating its potential as a drug-like molecule, adhering to general drug development guidelines. Additionally, its low PAINS Alter value suggested a lower risk of being a promiscuous compound, making it more suitable for drug development. Moreover, the higher SA Score of Rhapontin indicated relatively facile synthesis, which facilitated further research. Furthermore, the higher proportion of sp3-hybridized carbon atoms (Wei et al., 2020) in Rhapontin suggested its potential for enhanced drug activity. Notably, the receptor selectivity analyses, including docking scoring, ligand efficiency, and ligand strain energy, demonstrated that Rhapontin exhibited exceptional selectivity against FGFR3, as depicted in Figures 4E,F.

**TABLE 3:** Medicinal chemistry of 3 compounds.

Compound name	Rhapontin	PD173074	Isoquercetin
QED	0.366	0.347	0.229
SA Score	3.763	3.634	4.008
FSP^3^	0.333	0.5	0.286
MCE-18	67.143	22	91
PAINS Alter	0	0	1
Lipinski Rule	Accepted	Accepted	Rejected
Pfizer Rule	Accepted	Accepted	Accepted

In contrast, PD173074, while displaying some drug-like characteristics, exhibited a relatively lower QED value (Lipinski, 2004). Although it met the criteria of the Pfizer Rule, its performance might not be as effective as Rhapontin in certain aspects. The smaller Molar Refractivity (MCE-18) (Ivanenkov et al., 2019) value of PD173074 suggested a smaller molecular volume, potentially affecting interactions within the biological system. Despite having a PAINS Alter value of 0, indicating a lower probability of being a promiscuous compound, further investigation was still warranted.

Regarding Isoquercetin, its lower QED value indicated the necessity for further optimization. While satisfying the Pfizer Rule, its PAINS Alter value of 1 implied potential promiscuity, demanding additional evaluation. Isoquercetin’s higher SA Score indicated relatively facile synthesis, but its larger MCE-18 value suggested it might occupy a larger volume during interactions.

### 3.5 Exploring ligand binding effects on FGFR3 flexibility and interactions based om RMSF and interaction analysis

Through molecular dynamics simulations, studying the interactions between receptors and ligands provides in-depth insights into the binding modes and dynamic processes of drugs with their target receptors. This valuable information supports subsequent drug development, aiding in the optimization and improvement of drug molecules to enhance their affinity and selectivity towards target receptors, thus improving drug efficacy and safety, and providing scientific foundations for drug development.

**FIGURE 5:**
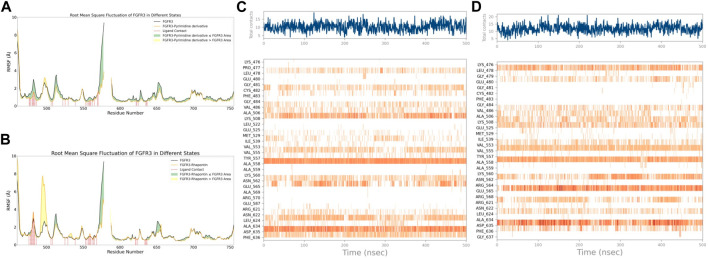
RMSF and Interaction Analysis. **(A)** RMSF changes of FGFR3 before and after binding with Pyrimidine Derivative 37b. **(B)** RMSF changes of FGFR3 before and after binding with Rhapontin. **(C)** Dynamic changes of Pyrimidine Derivative 37b′s contacts with FGFR3 residues over time. **(D)** Dynamic changes of Rhapontin’s contacts with FGFR3 residues over time.

With this purpose in mind, we first used the RMSF of FGFR3 in its apo state as a baseline to observe the similarities and differences in the effects of Pyrimidine Derivative 37b and Rhapontin on FGFR3, as shown in Figures 5A,B. Comparatively, the main differences between the two ligands were observed in two peptide segments. Firstly, in the region of 491–500, both Pyrimidine Derivative 37b and Rhapontin increased the flexibility to varying degrees, with Rhapontin causing a significantly greater effect. Secondly, in the region of 600–640, Pyrimidine Derivative 37b did not exhibit any significant influence, while Rhapontin slightly increased the flexibility in this area. Apart from these differences, both ligands showed minimal distinctions in their overall impact on FGFR3 residues and their contact frequency with FGFR3 residues.

Regarding the contact situation with residues, as shown in Figures 5C,D, the overall pattern was quite similar, but the average contact frequency in the simulation was higher for Rhapontin than for Pyrimidine Derivative 37b. For a detailed comparison of the binding conformations of Rhapontin before and after simulation, please refer to Supplementary Figure S1. However, this did not appear to be due to unreasonable conformations of the compounds inside the binding pocket but rather an increased contact frequency with certain residues, such as Glu-565 and Asp-635, which showed higher interaction frequencies than Rhapontin. The upregulation of RMSF in the region of 491–500 might not be directly related to changes in contact frequency with Rhapontin, as neither Pyrimidine Derivative 37b nor Rhapontin directly contacts these residues. A plausible explanation could be that Rhapontin does not contact residues 482 and 483, indirectly relieving the restrictions on this peptide segment.

**FIGURE 6:**
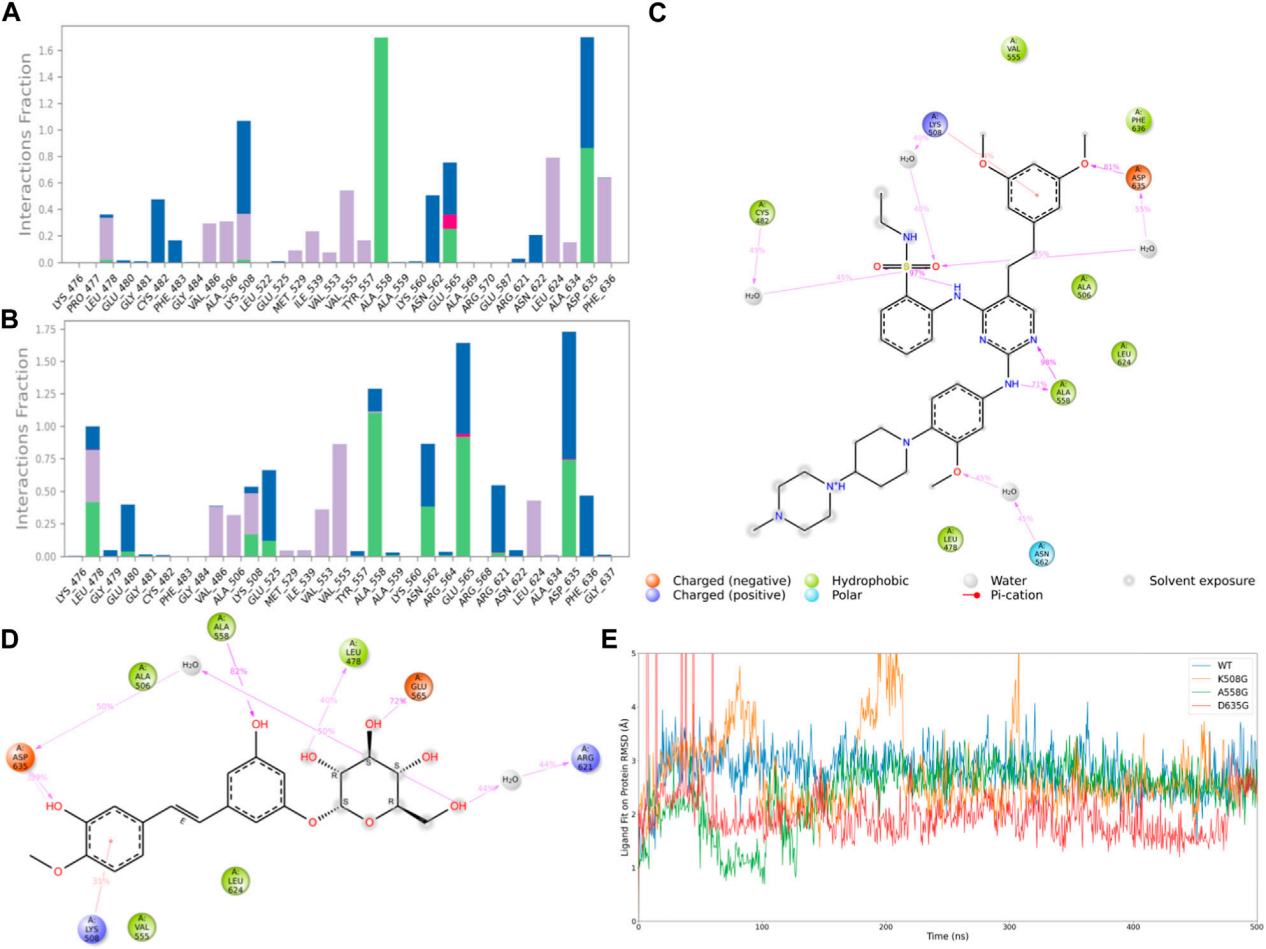
Protein-Ligand Interactions and Contact Analysis of 2 Compounds. **(A,B)** Columnar Statistical Analysis of Interaction between Pyrimidine Derivative 37b and Rhapontin **(C)** ligand-protein interactions of Pyrimidine Derivative 37b **(D)** ligand-protein interactions of Rhapontin **(E)** The binding stability of the receptor and ligand changes after Rhapontin binds to different FGFR3 mutants.

Subsequently, after classifying and statistically analyzing the interactions between compounds and individual residues, we selected residues with interaction frequencies exceeding 30% and depicted the interaction details between these residues and the compounds, as shown in Figures 6A–D. The interaction statistics showed consistency with the differences mentioned earlier, where the number of interacting residues with Rhapontin was fewer than with Pyrimidine Derivative 37b, but the interaction frequencies were slightly higher. Furthermore, in the interaction detail plots, it was observed that three key residues in the FGFR3 pharmacophore relationship, Lys-508, Ala-558, and Asp-635, were reproduced in the interaction details with Rhapontin. To further validate the importance of these three sites in Rhapontin binding, we conducted dynamic simulations with the three sites mutated to Gly and assessed their impact on the binding between the two, as shown in Figure 6E. It was evident that all three mutations significantly affected the binding between Rhapontin and FGFR3, demonstrating the importance of these residues in Rhapontin binding.

### 3.6 Biological evaluation of rhapontin through CCK-8 assay

**FIGURE 7:**
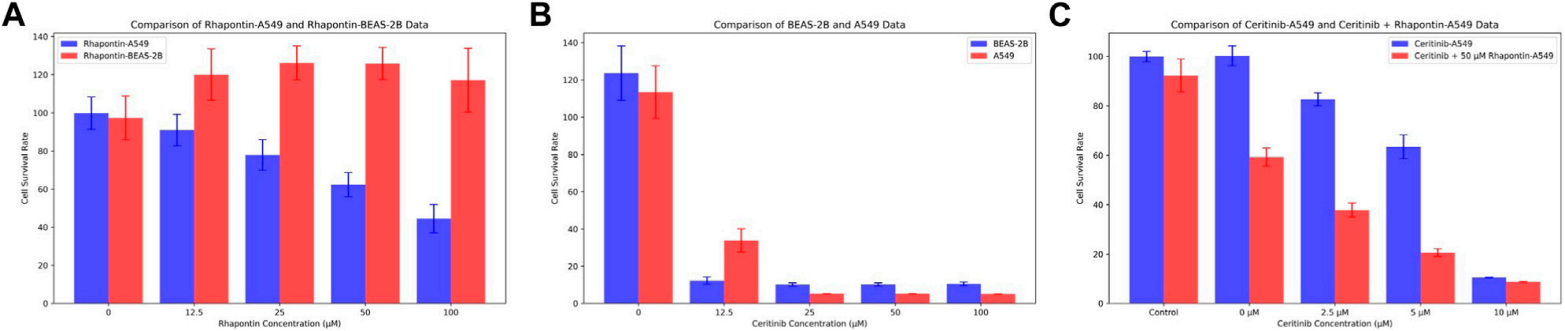
Verification of Rhapontin's anti-tumor activity and its sensitization effect on Ceritinib. **(A)** The effects of different concentrations of Rhapontin on the proliferation of A549 and BEAS-2B cells. **(B)** The effects of different concentrations of Ceritinib on the proliferation of A549 and BEAS-2B cells. **(C)** lThe effects of different concentrations of Ceritinib on A549 proliferation before and after combined use with Rhapontin.

In consideration of the limitations of our previous theoretical analyses, we conducted further experimental validations on Rhapontin. Initially, we subjected both BEAS-2B and A549 cell lines to varying concentrations of Rhapontin and Ceritinib (0–100 μM), as depicted in Figures 7A,B. As concentrations escalated, Rhapontin demonstrated a concentration-dependent proliferation inhibitory effect on A549 cells, with an IC50 of 62 μM. Despite its IC50 being significantly higher than that of Ceritinib, Rhapontin exhibited minimal impact on the proliferation of BEAS-2B cells. Conversely, Ceritinib exhibited a notable proliferation inhibitory effect on normal cells, including instances of substantial cytotoxicity.

Considering the potential of FGFR3 inhibitors to sensitize Ceritinib, we subsequently employed a reduced concentration (50 μM) of Rhapontin in combination with varying doses of Ceritinib (0–10 μM, adjusted from previous concentrations). The outcomes, as illustrated in Figure 7C, indeed displayed an enhanced tumor inhibitory effect to a certain extent when distinct concentrations of Ceritinib were co-administered with 50 μM Rhapontin. This co-administration led to a heightened sensitization of A549 cells to Ceritinib.

## 4 Discussion

The present study presents a systematic exploration aimed at augmenting the efficacy of Ceritinib, a prominent FGFR3 inhibitor, via the integration of natural compound-derived alternatives. Our investigation embraces a multidimensional approach, employing active learning derived virtual screen (Ma et al., 2009), deep learning derived QSAR modeling (Matsuzaka and Uesawa, 2023), molecular dynamics simulations (Duay et al., 2023), and biological assays to dissect the mechanisms underlying the potential synergy between Ceritinib and the identified natural compounds.

The selection of natural compounds as potential drug candidates draws attention to their inherent structural diversity and recognized pharmacological safety (Zhang et al., 2023). Natural products have, over the years, emerged as a wellspring of bioactive molecules, often possessing unique chemical scaffolds and physiological properties (Safranko et al., 2023). Notably, the prospect of leveraging certain natural compounds as nutraceutical agents underscores their compatibility with biological systems and augments the overall therapeutic potential (Wang and Wang, 2021).

Rhapontin, one of the highlighted natural compounds, presents intriguing prospects despite its moderate inhibitory activity in comparison to Pyrimidine Derivative 37b. This finding resonates with the broader paradigm of molecular design, urging for meticulous structural optimization to fine-tune both binding interactions and inhibitory potency (Azimian and Dastmalchi, 2023). The journey toward harnessing Rhapontin’s full potential entails a systematic exploration of its structural landscape, with a focus on judicious modifications to enhance its binding interactions.

The combined application of Rhapontin and Ceritinib, while not achieving the zenith of efficacy exhibited by certain established combination therapies, merits profound scrutiny (Krol et al., 2023). The nuanced response could stem from intricate intracellular interactions, wherein Rhapontin’s engagement with alternative molecular targets competes with its interaction with FGFR3 (Ho et al., 2014; Cascetta et al., 2022). This observation augments the need for a rigorous dissection of these competitive binding events, necessitating an iterative process of targeted compound engineering (Ho et al., 2014)

## 5 Conclusion

This study underscores the potential of natural compound-derived FGFR3 inhibitors to anti-cancer and sensitize Ceritinib. The utilization of natural compounds not only diversifies the drug discovery landscape but also accentuates their potential as bioactive agents with intrinsic safety profiles. Rhapontin’s modest inhibitory activity, coupled with its structural attributes, calls for a deeper exploration to unlock its latent potential. The observed synergy between Ceritinib and Rhapontin, albeit nuanced, underscores the intricate cellular dynamics that govern combination therapies. As we continue to unravel the complexities of molecular interactions, strategic compound engineering offers a promising avenue to enhance therapeutic outcomes and guide the evolution of precision medicine paradigms.

## Data Availability

The raw data supporting the conclusion of this article will be made available by the authors, without undue reservation.
