# Supplementary figures and images for: Active and machine learning-enhanced discovery of new FGFR3 inhibitor, Rhapontin, through virtual screening of receptor structures and anti-cancer activity assessment
Source: Front Mol Biosci. 2024 Jun 11;11:1413214. doi: 10.3389/fmolb.2024.1413214 (PMC11196408; doi:10.3389/fmolb.2024.1413214)

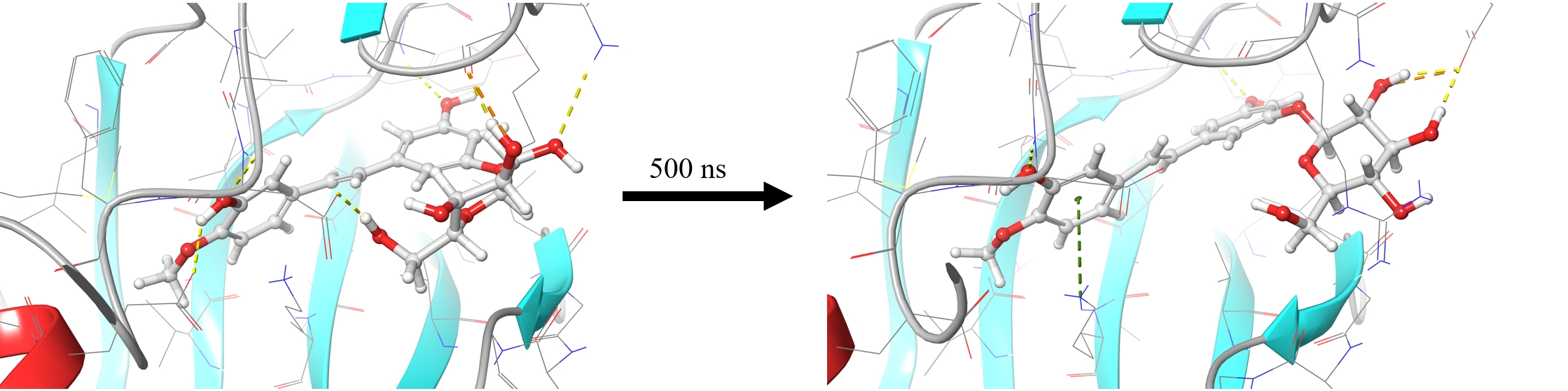

Supplement: Supplementary file 1 [file Image1.PNG]
